# Solid Lipid Nanoparticles: A Modern Formulation Approach in Drug Delivery System

**DOI:** 10.4103/0250-474X.57282

**Published:** 2009

**Authors:** S. Mukherjee, S. Ray, R. S. Thakur

**Affiliations:** Department of Pharmaceutics, Krupanidhi College of Pharmacy, Bangalore-560 034, India

**Keywords:** Solid lipid nanoparticles (SLN), colloidal drug carriers, homogenization, TEM, PCS, biodistribution, targeting

## Abstract

Solid lipid nanoparticles are at the forefront of the rapidly developing field of nanotechnology with several potential applications in drug delivery, clinical medicine and research, as well as in other varied sciences. Due to their unique size-dependent properties, lipid nanoparticles offer the possibility to develop new therapeutics. The ability to incorporate drugs into nanocarriers offers a new prototype in drug delivery that could be used for secondary and tertiary levels of drug targeting. Hence, solid lipid nanoparticles hold great promise for reaching the goal of controlled and site specific drug delivery and hence have attracted wide attention of researchers. This review presents a broad treatment of solid lipid nanoparticles discussing their advantages, limitations and their possible remedies. The different types of nanocarriers which were based on solid lipid like solid lipid nanoparticles, nanostructured lipid carriers, lipid drug conjugates are discussed with their structural differences. Different production methods which are suitable for large scale production and applications of solid lipid nanoparticles are described. Appropriate analytical techniques for characterization of solid lipid nanoparticles like photon correlation spectroscopy, scanning electron microscopy, differential scanning calorimetry are highlighted. Aspects of solid lipid nanoparticles route of administration and their biodistribution are also incorporated. If appropriately investigated, solid lipid nanoparticles may open new vistas in therapy of complex diseases.

Colloidal particles ranging in size between 10 and 1000 nm are known as nanoparticles. They are manufactured from synthetic/natural polymers and ideally suited to optimize drug delivery and reduce toxicity. Over the years, they have emerged as a variable substitute to liposomes as drug carriers. The successful implementation of nanoparticles for drug delivery depends on their ability to penetrate through several anatomical barriers, sustained release of their contents and their stability in the nanometer size. However, the scarcity of safe polymers with regulatory approval and their high cost have limited the wide spread application of nanoparticles to clinical medicine[1].

To overcome these limitations of polymeric nanoparticles, lipids have been put forward as an alternative carrier, particularly for lipophilic pharmaceuticals. These lipid nanoparticles are known as solid lipid nanoparticles (SLNs), which are attracting wide attention of formulators world-wide[2]. SLNs are colloidal carriers developed in the last decade as an alternative system to the existing traditional carriers (emulsions, liposomes and polymeric nanoparticles). They are a new generation of submicron-sized lipid emulsions where the liquid lipid (oil) has been substituted by a solid lipid. SLN offer unique properties such as small size, large surface area, high drug loading and the interaction of phases at the interfaces, and are attractive for their potential to improve performance of pharmaceuticals, neutraceuticals and other materials[3].

SLNs are attracting major attention as novel colloidal drug carrier for intravenous applications[1]. The SLNs are sub-micron colloidal carrier which is composed of physiological lipid, dispersed in water or in an aqueous surfactant solution. The Pubmed search till the date indicates the trends in SLN research, given in fig. 1. So if systematically investigated, SLNs may open new vista in research and therapy[4].

**Fig. 1:**
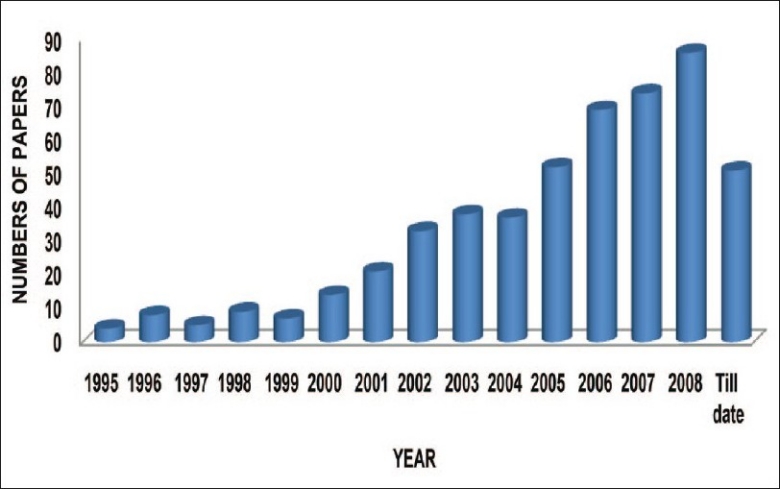
Trends in solid lipid nanoparticles research

## ADVANTAGES AND PROBLEMS OF SLNS AND OTHER NANOPARTICLES

SLNs combine the advantages and avoid the drawbacks of several colloidal carriers of its class, shows in Table 1[5,6]. Potential disadvantages such as poor drug loading capacity, drug expulsion after polymeric transition during storage and relatively high water content of the dispersions (70-99.9%) have been observed. The drug loading capacity of conventional SLN is limited by the solubility of drug in the lipid melt, the structure of the lipid matrix and the polymeric state of the lipid matrix. If the lipid matrix consists of especially similar molecules (i.e. tristearin or tripalmitin), a perfect crystal with few imperfections is formed. Since incorporated drugs are located between fatty acid chains, between the lipid layers and also in crystal imperfections, a highly ordered crystal lattice can not accommodate large amounts of drug. Therefore the use of more complex lipids is more sensible for higher drug loading.

**TABLE 1:** ADVANTAGES OF SOLID LIPID NANOPARTICLES

Advantages of solid lipid nanoparticles
Control and/or target drug release.
Improve stability of pharmaceuticals.
High and enhanced drug content (compared to other carriers).
Feasibilities of carrying both lipophilic and hydrophilic drugs.
Most lipids being biodegradable, SLNs have excellent biocompatibility.
Water based technology (avoid organic solvents).
Easy to scale-up and sterilize.
More affordable (less expensive than polymeric/surfactant based carriers).
Easier to validate and gain regulatory approval.

### Nanostructured lipid carriers (NLC):

NLC were introduced to overcome the potential difficulties with SLNs[7–9]. The goal was to increase the drug loading and prevent drug expulsion. This could be visualised in three ways. In the first model, spatially different lipids (like glycerides) composed of different fatty acids are mixed. The use of spatially different lipids leads to larger distances between the fatty acid chains of the glycerides and general imperfections in the crystal and thus provides more room for accommodation of guest molecules. The highest drug load could be achieved by mixing solid lipids with small amounts of liquid lipids (oils). This model is called imperfect type NLC. Drugs showing higher solubility in oils than in solid lipids can be dissolved in the oil and yet be protected from degradation by the surrounding solid lipids. These types of NLC are called multiple types NLC, and are analogous to w/o/w emulsions since it is an oil-in-solid lipid-in-water dispersion.

Since drug expulsion is caused by ongoing crystallization or transformation of the solid lipid, this can be prevented by the formation of a third type, the amorphous type NLC. Here the particles are solid but crystallization upon cooling is avoided by mixing special lipids like hydroxyl octacosanyl, hydroxyl stearate and isopropyl myristate. The NLCs have mainly been investigated in the topical and dermatological preparations[10] in the delivery of clotrimazole[11,12], ketoconazole[13], other antifungal imidazoles[14] and ascorbyl palmitate[9].

### Lipid drug conjugates (LDC):

A major problem of SLNs is the low capacity to load hydrophilic drugs due to partitioning effects during the production process. Only highly potent low dose hydrophilic drugs may be suitably incorporated in the solid lipid matrix[15]. In order to overcome this limitation, the so called LDC nanoparticles with drug loading capacities of up to 33% have been developed[10]. An insoluble drug-lipid conjugate bulk is first prepared either by salt formation (e.g. with a fatty acid) or by covalent linking (e.g. to ester or ethers). The obtained LDC is then processed with an aqueous surfactant solution (such as Tweens) to a nanoparticle formulation using high pressure homogenization (HPH). Such matrices may have potential application in brain targeting of hydrophilic drugs in serious protozoal infections[16].

## SLN PREPARATION

SLNs are made up of solid lipid, emulsifier and water/solvent (Table 2). The lipids used may be triglycerides (tri-stearin), partial glycerides (Imwitor), fatty acids (stearic acid, palmitic acid), and steroids (cholesterol) and waxes (cetyl palmitate). Various emulsifiers and their combination (Pluronic F 68, F 127) have been used to stabilize the lipid dispersion. The combination of emulsifiers might prevent particle agglomeration more efficiently[3].

**TABLE 2:** INGREDIENTS USED IN THE PREPARATION OF NANOPARTICLES

Name of the ingredients	Concentrations	Reference
Lipid	3.33% w/v	17
Phospholipids	0.6-1.5%	18
Glycerol	2-4%	--
Poloxamer 188	1.2-5% w/w	19
Soy phosphatidyl choline	95%	--
Compritol	10%	--
Cetyl palmitate	10% w/w	20
Tego care 450 (surfactant)	1.2% w/w	--
PEG 2000	0.25%	--
PEG 4500	0.5%	--
Tween 85	0.5%	21
Ethyl oleate	30%	--
Na alginate	70%	--
Ethanol/butanol	2%	22
Tristearin glyceride	95%	18
PEG 400	5%	--
Isopropyl myristate	3.60%	--
Pluronic F 68	40%	--
Tween 80	50%	21

A clear advantage of SLN is the fact that the lipid matrix is made from physiological lipids which decreases the danger of acute and chronic toxicity. The choice of the emulsifier depends on the administration route with a suitable number of emulsifier suitable for parenteral administration. Different methods of SLNs preparation are given in Table 3.

**TABLE 3:** METHODS OF SLN PREPARATION

Different methods of SLN preparation	Reference
High shear homogenization:	23-25
Hot homogenization	26,27
Cold homogenization	28
Ultrasonication/high speed homogenization:	29,30
Probe ultrasonication	
Bath ultrasonication	
Solvent emulsification/evaporation	31,32
Micro emulsion based SLN preparations	33-37
SLN preparation by using supercritical fluid	38-40
Spray drying method	41
Double emulsion method	42

## METHOD OF SLN PREPARATION

### High shear homogenization:

High shear homogenization technique were initially used for the production of solid lipid nanodispersions[17,18]. Both methods are widespread and easy to handle. However, dispersion quality is often compromised by the presence of micro particles. High-speed homogenization method is used to produce SLN by melt emulsification[19]. Olbrich *et al*. investigated the influence of different process parameters, including emulsification time, stirring rate and cooling condition on the particle size and zeta potential. Lipids used in this study included trimyristin, tripalmitin, a mixture of mono, di and triglycerides (Witepsol W35, Witepsol H35) with glycerol behenate and poloxamer 188 used as steric stabilizers (0.5% w/w). For Witepsol W35 dispersions the best SLN quality was obtained after stirring for 8 min at 20,000 rpm followed by cooling 10 min and stirring at 5000 rpm at a room temp. In contrast, the best conditions for Dynasan116 dispersions were a 10-min emulsification at 25,000 rpm and 5 min of cooling at 5,000 rpm in cool water (≈16°)[16]. Higher stirring rates did not significantly change the particle size, but slightly improved the polydispersity index.

### Hot homogenization:

Hot homogenization is carried out at temperatures above the melting point of the lipid and is similar to the homogenization of an emulsion. A pre-emulsion of the drug loaded lipid melt and the aqueous emulsifier phase (same temperature) is obtained by high-shear mixing device (like silversion-type homogenizer). The quality of the pre-emulsion affects the quality of the final product to a great extent and it is desirable to obtain droplets in the size range of a few micrometers. High pressure homogenization of the pre-emulsion is done above the lipid melting point. Usually, lower particle sizes are obtained at higher processing temperatures because of lowered viscosity of the lipid phase[20], although this might also accelerate the drug and carrier degradation. Better products are obtained after several passes through the high-pressure homogenizer (HPH), typically 3-5 passes. High pressure processing always increases the temperature of the sample (approximately 10° at 500 bar)[21]. In most cases, 3-5 homogenization cycles at 500-1500 bar are sufficient. Increasing the homogenization leads to an increase of the particle size due to particle coalescence, this occurs because of the high kinetic energy of the particles.

### Cold homogenization:

The cold homogenization process is carried out with the solid lipid and therefore is similar to milling of a suspension at elevated pressure. To ensure the solid state of the lipid during homogenization, effective temperature regulation is needed[21]. Cold homogenization has been developed to overcome the following problems of the hot homogenization technique such as: Temperature mediated accelerated degradation of the drug payload, Partitioning and hence loss of drug into the aqueous phase during homogenization, Uncertain polymorphic transitions of the lipid due to complexity of the crystallization step of the nanoemulsion leading to several modifications and/or super cooled melts.

The first preparatory step is the same as in the hot homogenization procedure and includes the solubilization or dispersion of the drug in the lipid melt. However, the subsequent steps differ. The drug containing melt is cooled rapidly (using dry ice or liquid nitrogen) to favor homogenous drug distribution in the lipid matrix. In effect, the drug containing solid lipid is pulverized to micropaticles by ball/mortar milling. Typical particle sizes attained are in the range 50-100 microns. Chilled processing further facilitated particle milling by increasing the lipid fragility. The SLNs are dispersed in a chilled emulsifier solution. The dispersion is subjected to high pressure homogenization at or below room temperature with appropriate temperature control keeping in view the usual rise in temperature during high pressure processing. However, compared to hot homogenization, larger particle sizes and a broader size distribution are typical of cold homogenized samples[22]. The method of cold homogenization minimizes the thermal exposure of the sample, but it does not avoid it due to the melting of the lipid/drug mixture in the initial step.

### Ultrasonication or high speed homogenization:

SLN were also developed by high speed stirring or sonication[23,24]. A most advantages are that, equipments that are used here are very common in every lab. The problem of this method is broader particle size distribution ranging into micrometer range. This lead physical instability likes particle growth upon storage. Potential metal contamination due to ultrasonication is also a big problem in this method. So for making a stable formulation, studies have been performed by various research groups that high speed stirring and ultrasonication are used combined and performed at high temperature.

### SLN prepared by solvent emulsification/evaporation:

For the production of nanoparticle dispersions by precipitation in o/w emulsions[25] the lipophilic material is dissolved in water-immiscible organic solvent (cyclohexane) that is emulsified in an aqueous phase. Upon evaporation of the solvent nanoparticle dispersion is formed by precipitation of the lipid in the aqueous medium. The mean diameter of the obtained particles was 25 nm with cholesterol acetate as model drug and lecithin/sodium glycocholate blend as emulsifier. The reproducibility of the result was confirmed by Siekmann and Westesen, who produced the cholesterol acetate nanoparticles of mean size 29 nm[26].

### Micro emulsion based SLN preparations:

Gasco and co-workers developed SLN preparation techniques which are based on the dilution of microemulsions[27]. They are made by stirring an optically transparent mixture at 65-70° which is typically composed of a low melting fatty acid (stearic acid), an emulsifier (polysorbate 20, polysorbate 60, soy phosphatidylcholine, and sodium taurodeoxycholate), co-emulsifiers (sodium monooctylphosphate) and water. The hot microemulsion is dispersed in cold water (2-3°) under stirring. Typical volume ratios of the hot microemulsion to cold water are in the range of 1:25 to 1:50. The dilution process is critically determined by the composition of the microemulsion. According to the literature[28,29], the droplet structure is already contained in the microemulsion and therefore, no energy is required to achieve submicron particle sizes. With respect to the similarities of the production procedure of polymer nanoparticles described by French scientists, different mechanisms might be considered. Fessi produced polymer particles by dilution of polymer solutions in water. According to De Labouret *et al.*[30], the particle size is critically determined by the velocity of the distribution processes. Nanoparticles were produced only with solvents which distribute very rapidly into the aqueous phase (acetone), while larger particle sizes were obtained with more lipophilic solvents. The hydrophilic co-solvents of the microemulsion might play a similar role in the formation of lipid nanoparticles as the acetone for the formation of polymer nanoparticles[31].

### SLN preparation by using supercritical fluid:

This is a relatively new technique for SLN production and has the advantage of solvent-less processing[32,33]. There are several variations in this platform technology for powder and nanoparticle preparation. SLN can be prepared by the rapid expansion of supercritical carbon dioxide solutions (RESS) method. Carbon dioxide (99.99%) was the good choice as a solvent for this method[34].

### Spray drying method:

It's an alternative procedure to lyophilization in order to transform an aqueous SLN dispersion into a drug product. It's a cheaper method than lyophilization. This method cause particle aggregation due to high temperature, shear forces and partial melting of the particle. Freitas and Mullera[35] recommends the use of lipid with melting point >70° for spray drying. The best result was obtained with SLN concentration of 1% in a solution of trehalose in water or 20% trehalose in ethanol-water mixtures (10/90 v/v).

### Double emulsion method:

For the preparation of hydrophilic loaded SLN, a novel method based on solvent emulsification-evaporation has been used[36]. Here the drug is encapsulated with a stabilizer to prevent drug partitioning to external water phase during solvent evaporation in the external water phase of w/o/w double emulsion.

## CHARACTERIZATION OF SLN QUALITY AND STRUCTURE

Adequate and proper characterization of the SLNs is necessary for its quality control. However, characterization of SLN is a serious challenge due to the colloidal size of the particles and the complexity and dynamic nature of the delivery system. The important parameters which need to be evaluated for the SLNs are, particle size, size distribution kinetics (zeta potential), degree of crystallinity and lipid modification (polymorphism), coexistence of additional colloidal structures (micelles, liposome, super cooled, melts, drug nanoparticles), time scale of distribution processes, drug content, *in vitro* drug release and surface morphology.

The particle size/size-distribution may be studied using photon correlation spectroscopy (PCS), transmission electron microscopy (TEM), scanning electron microscopy (SEM), atomic force microscopy (AFM), scanning tunneling microscopy (STM), or freeze fracture electron microscopy (FFEM).

### Measurement of particle size and zeta potential[37]:

Photon correlation spectroscopy (PCS) and laser diffraction (LD) are the most powerful techniques for routine measurements of particle size. The Coulter method is rarely used to measure SLN particle size because of difficulties in the assessment of small nanoparticle and the need of electrolytes which may destabilize colloidal dispersions. PCS (also known dynamic light scattering) measures the fluctuation of the intensity of the scattered light which is caused by the particle movement. This method covers a size range from a few nanometers to about 3 microns. This means that PCS is a good tool to characterize nanoparticles, but it is not able to detect larger microparticles. They can be visualized by means of LD measurements. This method is based on the dependence of the diffraction angle on the particle radius (Fraunhofer spectra). Smaller particles cause more intense scattering at high angles compared to the larger ones. A clear advantage of LD is the coverage of a broad size range from the nanometer to the lower millimeter range. The development of polarization intensity differential scattering (PIDS) technology greatly enhanced the sensitivity of LD to smaller particles. However, despite this progress, it is highly recommended to use PCS and LD simultaneously. It should be kept in mind that both methods do not ‘measure’ particle size. Rather, they detect light scattering effects which are used to calculate particle size. For example, uncertainties may result from non–spherical particle shapes. Platelet structures commonly occur during lipid crystallization and have also been suggested in the SLN. Further, difficulties may arise both in PCS and LD measurements for samples which contain several populations of different size. Therefore, additional techniques might be useful. For example, light microscopy is recommended, although it is not sensitive to the nanometer size range. It gives a fast indication of the presence and character of microparticles (microparticles of unit form or microparticles consisting of aggregates of smaller particles). Electron microscopy provides, in contrast to PCS and LD, direct information on the particle shape. However, the investigator should pay special attention to possible artifacts which may be caused by the sample preparation. For example, solvent removal may cause modifications which will influence the particle shape. Zeta potential is an important product characteristic of SLNs since its high value is expected to lead to deaggregation of particles in the absence of other complicating factors such as steric stabilizers or hydrophilic surface appendages. It is usually measured by zetameter.

### Dynamic light scattering (DLS):

DLS, also known as PCS or quasi-elastic light scattering (QELS) records the variation in the intensity of scattered light on the microsecond time scale. This variation results from interference of light scattered by individual particles under the influence of Brownian motion, and is quantified by compilation of an autocorrelation function. This function is fit to an exponential, or some combination or modification thereof, with the corresponding decay constant(s) being related to the diffusion coefficient(s)[37]. Using standard assumptions of spherical size, low concentration, and known viscosity of the suspending medium, particle size is calculated from this coefficient. The advantages of the method are the speed of analysis, lack of required calibration, and sensitivity to submicrometer particles.

### Static light scattering/Fraunhofer diffraction:

Static light scattering (SLS) is an ensemble method in which the pattern of light scattered from a solution of particles is collected and fit to fundamental electromagnetic equations in which size is the primary variable. The method is fast and rugged, but requires more cleanliness than DLS, and advance knowledge of the particles' optical qualities.

### Acoustic methods:

Another ensemble approach, acoustic spectroscopy, measures the attenuation of sound waves as a means of determining size through the fitting of physically relevant equations. In addition, the oscillating electric field generated by the movement of charged particles under the influence of acoustic energy can be detected to provide information on surface charge.

### Nuclear magnetic resonance (NMR):

NMR can be used to determine both the size and the qualitative nature of nanoparticles. The selectivity afforded by chemical shift complements the sensitivity to molecular mobility to provide information on the physicochemical status of components within the nanoparticle.

### Electron microscopy[37]:

SEM and TEM provide a way to directly observe nanoparticles, physical characterization of nanoparticles with the former method being better for morphological examination. TEM has a smaller size limit of detection, is a good validation for other methods, and affords structural required, and one must be cognizant of the statistically small sample size and the effect that vacuum can have on the particles.

### Atomic force microscopy (AFM)[38]:

In this technique, a probe tip with atomic scale sharpness is rastered across a sample to produce a topological map based on the forces at play between the tip and the surface. The probe can be dragged across the sample (contact mode), or allowed to hover just above (noncontact mode), with the exact nature of the particular force employed serving to distinguish among the subtechniques. That ultrahigh resolution is obtainable with this approach, which along with the ability to map a sample according to properties in addition to size, e.g., colloidal attraction or resistance to deformation, makes AFM a valuable tool.

### X-ray diffraction (powder X-ray diffraction) and differential scanning calorimetry (DSC):

The geometric scattering of radiation from crystal planes within a solid allow the presence or absence of the former to be determined thus permitting the degree of crystallinity to be assessed. Another method that is a little different from its implementation with bulk materials, DSC can be used to determine the nature and speciation of crystallinity within nanoparticles through the measurement of glass and melting point temperatures and their associated enthalpies[38].

## STERILIZATION OF SLNS

For intravenous and ocular administration SLN must be sterile. The high temperature reach during sterilization by autoclaving presumably causes a hot o/w microemulsion to form in the autoclave, and probably modifies the size of the hot nanodroplets. On subsequent slow cooling, the SLN reformed, but some nanodroplets may coalesce, producing larger SLN than the initial ones. Since SLN are washed before sterilization, amounts of surfactant and cosurfactant present in the hot system are smaller, so that the nanodroplets may be not sufficiently stabilized.

## ORAL LIPID BASED FORMULATIONS[39]

Among the benefits which oral lipid-based formulations can provide are included: Improvement and reduction in the variability of GI absorption of poorly water-soluble, lipophilic drugs. Possible reduction in, or elimination of, a number of development and processing steps (salt selection or identification of a stable crystalline form of the drug, coating, tastemasking, and reduced need for containment and clean-up requirements during manufacture of highly-potent or cytotoxic drug products). Reduction or elimination of positive food effect. Relative ease of manufacture using readily available equipment. Different types of oral lipid based formulation are like, single-component lipid solutions, self-emulsifying formulations, self-emulsifying solid dispersion formulations and melt pelletization. It has been revealed that the most frequently chosen excipients for preparing oral lipid-based formulations were dietary oils composed of medium (coconut or palm seed oil) or long-chain triglycerides (corn, olive, peanut, rapeseed, sesame, or soybean oils, including hydrogenated soybean or vegetable oils), lipid soluble solvents (polyethylene glycol 400, ethanol, propylene glycol, glycerin), and various pharmaceutically- acceptable surfactants (Cremophor® EL, RH40 or RH60; polysorbate 20 or 80; D-α-tocopherol polyethylene glycol 1000 succinate (TPGS®); Span 20; various Labrafils®, Labrasol®, and Gelucires®). These formulations, which took the form of either bulk oral solutions or liquid-filled hard or soft gelatin capsules, were applied in instances where conventional approaches (solid wet or dry granulation, or water-miscible solution in a capsule) did not provide sufficient bioavailability, or in instances in which the drug substance itself was an oil (dronabinol, ethyl icosapentate, indometacin farnesil, teprenone, and tocopherol nicotinate). The total daily drug dose administered in these formulations, which range in complexity from simple solutions of the drug in a dietary oil up to multi-excipient, self-emulsifying drug delivery systems (SEDDS), range from less than 0.25 μg to greater than 2000 mg. The amount of drug contained in a unit-dose capsule product ranges from 0.25 μg to 500 mg and for oral solution products, from 1 μg/ml to 100 mg/ml. The total amount of lipid excipient administered in a single dose of a capsule formulation ranges from 0.5 to 5 g, but can range from as low as 0.1 ml to as high as 20 ml for oral solution products. Some of these products tolerate room temperature storage for only brief periods of time and require long-term storage at 2-8° due to chemical and/or physical stability issues.

## ROUTES OF ADMINISTRATION AND THEIR BIODISTRIBUTION

The *in vivo* fate of the solid lipid nanoparticles will depend mainly on the administration route and distribution process (adsorption of biological material on the particle surface and desorption of SLN components into the biological surrounding). SLN are composed of physiological or physiologically related lipids or waxes. Therefore, pathways for transportation and metabolism are present in the body which may contribute to a large extent to the *in vivo* fate of the carrier. Probably the most important enzymes of SLN degradation are lipases, which are present in various organs and tissues. Lipases split the ester linkage and form partial glycerides or glycerol and free fatty acids. Most lipases require activation by an oil/water interface, which opens the catalytic center (lid opening). *In vitro* experiment indicates that solid lipid nanoparticles show different degradation velocities by the lipolytic enzyme pancreatic lipase as a function of their composition (lipid matrix, stabilizing surfactant)[40].

### Per oral administration:

Per oral administration forms of SLN may include aqueous dispersions or SLN-loaded traditional dosage forms such as tablets, pellets or capsules. The microclimate of the stomach favors particle aggregation due to the acidity and high ionic strength. It can be expected, that food will have a large impact on SLN performance, however no experimental data have been publish on this issue to our knowledge. The question concerning the influence of the gastric and pancreatic lipases on SLN degradation *in vivo* remains open, too. Unfortunately, only few *in vivo* studies have been performed yet.

### Parenteral administration:

SLN have been administered intravenously to animals. Pharmacokinetic studies of doxorubicin incorporated into SLN showed higher blood levels in comparison to a commercial drug solution after i.v. injection in rats. Regarding distribution, SLN were found to have higher drug concentrations in lung, spleen and brain, while the solution led to more distribution into liver and kidneys[41].

Yang *et al*. reported on the pharmacokinetics and body distribution of camptothecin after i.v. injection in mice. In comparison to a drug solution SLN was found to give much higher AUC/dose and mean residence times (MRT) especially in brain, heart and reticuloendothelial cells containing organs. The highest AUC ratio of SLN to drug solution among the tested organs was found in the brain[41,42].

### Transdermal application:

The smallest particle sizes are observed for SLN dispersions with low lipid content (up to 5%). Both the low concentration of the dispersed lipid and the low viscosity are disadvantageous for dermal administration. In most cases, the incorporation of the SLN dispersion in an ointment or gel is necessary in order to achieve a formulation which can be administered to the skin. The incorporation step implies a further reduction of the lipid content of the SLN dispersion resulting in semisolid, gel-like systems, which might be acceptable for direct application on the skin[43].

## APPLICATIONS

Solid lipid Nanoparticles possesses a better stability and ease of upgradability to production scale as compared to liposomes. This property may be very important for many modes of targeting. SLNs form the basis of colloidal drug delivery systems, which are biodegradable and capable of being stored for at least one year. They can deliver drugs to the liver *in vivo* and *in vitro* to cells which are actively phagocytic. There are several potential applications of SLNs some of which are given below:

### SLNs as gene vector carrier:

SLN can be used in the gene vector formulation[44]. In one work, the gene transfer was optimized by incorporation of a diametric HIV-1 HAT peptide (TAT 2) into SLN gene vector. There are several recent reports of SLN carrying genetic/peptide materials such as DNA, plasmid DNA and other nucleic acids[45,46]. The lipid nuclic acid nanoparticles were prepared from a liquid nanophase containing water and a water miscible organic solvent where both lipid and DNA are separately dissolved by removing the organic solvent, stable and homogeneously sized lipid-nuclic acid nanoparticle (70-100 nm) were formed. It's called genospheres. It is targeted specific by insertion of an antibody-lipo polymer conjugated in the particle.

### SLNs for topical use:

SLNs and NLCs have been used for topical application[10] for various drugs such as tropolide[47], imidazole antifungals[14], anticancers[48], vitamin A[49], isotretinoin[50], ketoconazole[51], DNA[52], flurbiprofen[53] and glucocorticoids[54]. The penetration of podophyllotoxin-SLN into stratum corneum along with skin surface lead to the epidermal targeting[48]. By using glyceryl behenate, vitamine A-loaded nanoparticles can be prepared. The methods are useful for the improvement of penetration with sustained release[49]. The isotretinoin-loaded lipid nanoparticles was formulated for topical delivery of drug. The soyabean lecithin and Tween 80 are used for the hot homogenization method for this. The methodology is useful because of the increase of accumulative uptake of isotretinoin in skin[50]. Production of the flurbiprofen-loaded SLN gel for topical application affer a potential advantages of delivering the drug directly to the site of action, which will produce higher tissue concentrations. Polyacrylamide, glycerol and water were used for the preparation of this type of SLN gel[52].

### SLNs as cosmeceuticals:

The SLNs have been applied in the preparation of sunscreens and as an active carrier agent for molecular sunscreens and UV blockers[54]. The *in vivo* study showed that skin hydration will be increased by 31% after 4 weeks by addition of 4% SLN to a conventional cream[55]. SLN and NLCs have proved to be controlled release innovative occlusive topicals[56]. Better localization has been achieved for vitamin A in upper layers of skin with glyceryl behenate SLNs compared to conventional formulations[57].

### SLNs for potential agriculture application:

Essential oil extracted from *Artemisia arboreseens* L when incorporated in SLN, were able to reduce the rapid evaporation compared with emulsions and the systems have been used in agriculture as a suitable carrier of ecologically safe pesticides[58]. The SLN were prepared here by using compritol 888 ATO as lipid and poloxamer 188 or Miranol Ultra C32 as surfactant.

### SLNs as a targeted carrier for anticancer drug to solid tumors:

SLNs have been reported to be useful as drug carriers to treat neoplasms[59]. Tamoxifen, an anticancer drug incorporated in SLN to prolong release of drug after i.v. administration in breast cancer and to enhance the permeability and retention effect[60]. Tumour targeting has been achieved with SLNs loaded with drugs like methotrexate[61] and camptothecin[62].

### SLNs in breast cancer and lymph node metastases:

Mitoxantrone-loaded SLN local injections were formulated to reduce the toxicity and improve the safety and bioavailability of drug[63]. Efficacy of doxorubicin (Dox) has been reported to be enhanced by incorporation in SLNs[64]. In the methodology the Dox was complexed with soybean-oil-based anionic polymer and dispersed together with a lipid in water to form Dox-loaded solid lipid nanoparticles. The system is enhanced its efficacy and reduced breast cancer cells.

### Oral SLNs in antitubercular chemotherapy:

Antitubercular drugs such as rifampicin, isonizide, pyrazinamide-loaded SLN systems, were able to decrease the dosing frequency and improve patient compliance[65]. By using the emulsion solvent diffusion technique this antitubercular drug loaded solid lipid nanoparticles are prepared. The nebulization in animal by incorporating the above drug in SLN also reported for improving the bioavailability of the drug[66].

### Stealth nanoparticles:

These provide a novel and unique drug-delivery system they evade quick clearance by the immune system. Theoretically, such nanoparticles can target specific cells. Studies with antibody labelled stealth lipobodies have shown increased delivery to the target tissue in accessible sites. Stealth SLNs have been successfully tested in animal models with marker molecules and drugs[67].

## CONCLUSIONS

In the early days of the 20^th^ century, Paul Ehrlich envisioned his magic bullet concept; the idea that drugs reaches the right site in the body, at the right time, at right concentration. It should not exert side effects, neither on its way to the therapeutic target, nor at the target site, nor during the clearance process. The SLNs have the potential to achieve, at least partially, these broad objectives. Apart from these, the regular objective of controlled drug delivery is aptly achieved with SLNs. They are relatively young drug delivery systems, having received primary attention from the early 1990s and future holds great promise for its systematic investigation and exploitation. We can expect many patented dosage forms in the form of SLNs in the future.
